# Replication study of SNP associations for colorectal cancer in Hong Kong Chinese

**DOI:** 10.1038/sj.bjc.6605977

**Published:** 2010-12-21

**Authors:** J W Ho, S-c Choi, Y-f Lee, T C Hui, S S Cherny, M-M Garcia-Barceló, L Carvajal-Carmona, R Liu, S-h To, T-k Yau, C C Chung, C C Yau, S M Hui, P Y Lau, C-h Yuen, Y-w Wong, S Ho, S S Fung, I P Tomlinson, R S Houlston, K K Cheng, P C Sham

**Affiliations:** 1 Department of Surgery, The University of Hong Kong, Pokfulam, Hong Kong; 2 Department of Psychiatry, The University of Hong Kong, Pokfulam, Hong Kong; 3 Nuffield Department of Medicine, Molecular and Population Genetics, University of Oxford, Wellcome Trust Centre for Human Genetics, Oxford OX3 7BN, UK; 4 Department of Clinical Oncology, Queen Mary Hospital, Pokfulam, Hong Kong; 5 Department of Surgery, Ruttonjee Hospital, 266 Queen's Road East, Wai Chai, Hong Kong; 6 Department of Clinical Oncology, Pamela Youde Nethersole Eastern Hospital, 3 Lok Man Road, Chai Wan, Hong Kong; 7 Department of Surgery, Pamela Youde Nethersole Eastern Hospital, 3 Lok Man Road, Chai Wan, Hong Kong; 8 Department of Clinical Oncology, Princess Margaret Hospital, 2-10 Princess Margaret Hospital Road, Lai Chi Kok, Kowloon, Hong Kong; 9 Department of Surgery, Princess Margaret Hospital, 2-10 Princess Margaret Hospital Road, Lai Chi Kok, Kowloon, Hong Kong; 10 Department of Surgery, Kwong Wah Hospital, 25 Waterloo Road, Kowloon, Hong Kong; 11 Department of Surgery, Tseung Kwan O Hospital, 2 Po Ning Lane, Hang Hau, Tseung Kwan O, New Territories, Hong Kong; 12 Department of Surgery, Tuen Mun Hospital, Tsing Chung Koon Road, Tuen Mun, New Territories, Hong Kong; 13 Section of Cancer Genetics, The Institute of Cancer Research, Brookes Lawley Building, Haddow Laboratories, Sutton, Surrey SM2 5 NG, UK; 14 Department of Public Health, Epidemiology and Biostatistics, The University of Birmingham, Public Health Building, Edgbaston, Birmingham B15 2TT, UK

**Keywords:** colorectal cancer, genetic, association, replication, Chinese

## Abstract

**Background:**

Recent genome-wide association studies of colorectal cancer (CRC) have identified common single-nucleotide polymorphisms (SNPs) mapping to 10 independent loci that confer modest increased risk. These studies have been conducted in European populations and it is unclear whether these observations generalise to populations with different ethnicities and rates of CRC.

**Methods:**

An association study was performed on 892 CRC cases and 890 controls recruited from the Hong Kong Chinese population, genotyping 32 SNPs, which were either associated with CRC in previous studies or are in close proximity to previously reported risk SNPs.

**Results:**

Twelve of the SNPs showed evidence of an association. The strongest associations were provided by rs10795668 on 10p14, rs4779584 on 15q14 and rs12953717 on 18q21.2. There was significant linear association between CRC risk and the number of independent risk variants possessed by an individual (*P*=2.29 × 10^−5^).

**Conclusion:**

These results indicate that some previously reported SNP associations also impact on CRC risk in the Chinese population. Possible reasons for failure of replication for some loci include inadequate study power, differences in allele frequency, linkage disequilibrium structure or effect size between populations. Our results suggest that many associations for CRC are likely to generalise across populations.

Colorectal cancer (CRC) affects over one million people each year worldwide (Tenesa and Dunlop, 2009). It is currently the third commonest malignancy and the fourth commonest cause of cancer-related mortality in the world (Stewart *et al*, 2003). The overall burden of the disease is set to increase further from the increasing incidence rates in Asian and African populations associated with the adoption of western diets (Tenesa and Dunlop, 2009). In Hong Kong, CRC is now the second commonest cancer (with 4084 cases in 2007) and the second commonest cause of cancer death (1690 deaths in 2007) (Hong Kong Cancer Registry, Hospital Authority, 2009).

Although dietary and lifestyle risk factors undoubtedly are major risk factors for CRC, twin studies have shown that ∼30% of the variation in susceptibility to CRC involves inherited genetic differences (Lichtenstein *et al*, 2000). However, high-penetrance susceptibility mutations account for <6% of CRC cases; the majority of inherited variance appearing to be a consequence of the co-inheritance of multiple low-risk variants (Lichtenstein *et al*, 2000; Bost *et al*, 2001).

Recent genome-wide association studies (GWAS) have provided statistically robust evidence for common susceptibility loci for CRC. These studies have so far identified common single-nucleotide polymorphisms (SNP) at 10 independent loci that confer modest increased risk to CRC (odds ratios (OR) ∼1.1–1.3) at 8q23.3, 8q24.21, 10p14, 11q23.1, 14q22.3, 15q13.3, 16q22.1, 18q21.1, 19q13.11 and 20p12.3 (Easton and Eeles, 2008; Le Marchand, 2009). These GWAS have been performed almost exclusively in populations of European ancestry, and the effects of these risk alleles in other populations are as yet unknown.

Understanding the effects of these variants in different populations is extremely important in terms of inferring the causality and mechanisms of colorectal tumourigenesis, as well as for the translation of these results to risk prediction in different populations. Colorectal cancer is a disease with very different incidence rates between populations (Curado *et al*, 2007). The risk variants may confer different magnitudes of increased risk in different populations for a variety of reasons, including differences in allele frequency and linkage disequilibrium (LD) structure, and difference in genetic and environmental backgrounds that interact with the variants (Sawyer *et al*, 2005; Weir *et al*, 2005; Ireland *et al*, 2006; Ioannidis, 2007).

To further our knowledge of the role of common genetic predisposition to CRC, we have examined the impact of the 10 known low-penetrance CRC risk loci in the Han Chinese population in Hong Kong using a case–control study design. We first examined variants, which were previously reported to have reached genome-wide significance (Broderick *et al*, 2007; Tomlinson *et al*, 2007, 2008; Houlston *et al*, 2008; Jaeger *et al*, 2008; Tenesa *et al*, 2008) for association with CRC risk, in an initial case–control sample. We then examined in an extended case–control series 22 additional SNPs, which have been associated with CRC risk in unpublished studies on European populations. Some of these SNPs were located close to SNPs genotyped in the first part of the study. Phase 1 can be regarded as a replication study of established associations in European populations, whereas Phase 2 is a replication study of more tentative associations as well as a more comprehensive screening of the risk loci evaluated in Phase 1.

## Materials and methods

### Subjects

Since October 2006, subjects (CRC cases and controls) have been recruited from seven departments of surgery and three departments of oncology in seven public hospitals in Hong Kong. The CRC cases were adults with histologically proven adenocarcinoma of the colon or rectum (international diseases 9 codes 153 and 154) diagnosed either (1) within 18 months before recruitment commencement date (prevalent cases) or (2) within the recruitment period (incident cases), treated at the seven participating hospitals. The controls were sex- and age-matched hospital inpatients or outpatients without a personal history of cancer or a family history of CRC in first-degree relatives treated at the participating hospitals.

Informed consent was obtained from all participants and the study protocol was approved by the Institution Review Boards of the seven participating hospitals in accordance with the declaration of Helsinki.

### Genotyping

Variation at 8q24.21, 10p14, 11q23.1, 14q22.3, 15q14, 16q22.1, 18q21.2, 19q12 and 20p12.3 loci was evaluated by genotyping cases and controls for rs6983267, rs7014346, rs706771, rs827401, rs7894531, rs7898455, rs4474353, rs10795668, rs3802842, rs11623717, rs17563, rs2071047, rs2761887, rs8014363, rs4444235, rs6494587, rs16969681, rs16970016, rs1554865, rs11632717, rs1406389, rs1919360, rs7165427, rs10318, rs4779584, rs9929218, rs12953717, rs4464148, rs4939827, rs10411210, rs961253 and rs355527.

DNA was extracted from EDTA-venous blood samples using standard methodology. The SNP genotyping was conducted using the Sequenom MassARRAY system (Sequenom, San Diego, CA, USA). Genotyping assays were designed using SpectroDESIGNER software version 2.0.0.17 (Sequenom). Quality control was monitored by including duplicate and four negative controls in each 384-well plate. Further quality control included the exclusion of SNPs with genotype call rates <95%, minor allele frequency (MAF) <5% and those that deviated significantly from Hardy–Weinberg equilibrium in the controls (*P*<0.01).

### Statistical and bioinformatic analysis

Haploview version 4.1 (Barrett *et al*, 2005) and HapMap CHP+JPT data (release 22; http://hapmap.ncbi.nlm.nih.gov/) was used to generate LD plots. The PLINK (Purcell *et al*, 2007) and R (Version 2.8.1; http://www.r-project.org/) were used for association analyses. The Cochran–Armitage trend test was used to examine association between CRC and SNP genotype (Armitage, 1995). In addition, logistic regression analysis of CRC on allele dosage (0, 1, 2) was performed, with adjustment for sex as covariate. Statistical significance was assessed on the basis of two-sided *P-*values, and allowance for multiple testing was made by using Bonferroni's correction and false discovery rates (FDR) methodology. Heterogeneity between the ORs in this study and those of previous studies was assessed by the Breslow–Day's test. Association between clinico-pathological variables and SNP genotype was analysed by the Armitage trend test or by logistic regression with sex as covariate, on the cases only. A composite score of genetic susceptibility was created from nine independent SNPs in Part 1, choosing only one SNP (the most significant) from each group of tightly linked SNPs. The composite score in an individual was calculated as the total number of high-risk alleles present in the individual (possible range 0–18). The association between the composite score and CRC risk was assessed by χ^2^ tests and by a Cochran–Armitage trend test.

## Results

In the first phase, we genotyped 716 CRC cases and 714 controls. The cases comprised 445 males and 271 females. In the second phase, an additional 176 cases and 180 controls were genotyped yielding a total of 892 cases and 890 controls. The clinical characteristics of the cases and controls are detailed in Table 1.

The 14 SNPs included in the first phase (rs6983267, rs7014346, rs10795668, rs3802842, rs4444235, rs4779584, rs10318, rs9929218, rs4939827, rs12953717, rs4464148, rs10411210 and rs355527) had an average genotyping call rate of 99.9% (Supplementary Table 1). One SNP, rs16893766, was monomorphic in this cohort and was thus not analysed. For the 22 SNPs included in the second phase, the overall genotyping call rates were 95.3%. Three SNPs were excluded from analysis because they had genotyping call rates <95% (rs133344771) or MAF <5% (rs11986063 and rs10424333). A total of 32 SNPs (13 from Phase 1 and 19 from Phase 2) annotating nine distinct loci provided data for the complete analysis. Ten SNPs were mapped to 15q, six SNPs each to 10p and 14q, three SNPs to 18q, two SNPs each to 8q and 20p and one SNP each to 11q, 16q and 19q.

Five of the 13 SNPs genotyped in Phase 1 were significantly associated with CRC risk (Table 2A). Although only the most significant SNP (rs10795668, *P*=0.0018) would be significant after Bonferroni's adjustment, all five nominally significant SNPs would be considered significant on a basis of an FDR of 0.1 (rs10795668, rs7014346, rs12953717, rs4779584 and rs4939827). For all five SNPs, the risk-increasing allele in this study is the same as in the original report of association. Two of the significant SNPs, rs4939827 and rs12953717 on Chromosome 18q21.2, are in strong LD with each other.

Seven of the 19 SNPs in Phase 2 were significant, but none were significant after Bonferroni's adjustment (Table 2B). All seven nominally significant SNPs would be considered significant at an FDR of 0.1 (rs7898455, rs4474353, rs7894531, rs1554865, rs16970016, rs706771 and rs827401). However, these significant SNPs are all in strong LD with SNPs significant in Part 1: rs7898455, rs4474353, rs7894531 rs706771 and rs827401 are in LD with rs10795668 on Chromosome 10p14, whereas rs16970016 and rs1554865 are in LD with rs4779584 on Chromosome 15q14.

In logistic regression analyses of SNPs within each LD region, the inclusion of additional SNPs to a model containing the most strongly associated SNP in each (i.e. rs10795668 on 10p14, rs4779584 on 15q14 and rs12953717 on 18q21.2) did not significantly improve the fit of the model, thus providing no evidence for more than one disease locus in each of these regions (Supplementary Table 2).

Collectively, these data are consistent with four independent CRC loci defined by SNPs rs10795668, rs12953717, rs4779584 and rs7014346.

**Figure 1:**
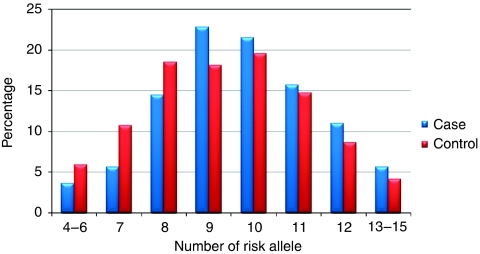
Composite index distribution in cases and controls.

In order to avoid bias, a composite index was calculated from all nine independent SNPs from Phase 1. This index was significantly associated with CRC risk (*P*_trend_=2.29 × 10^−5^) with an OR of over 2 for individuals with 12 or more high-risk alleles compared with individuals with 6 or fewer high-risk alleles (Table 3). The difference in composite index between cases and controls is shown graphically in Figure 1.

We assessed the associations between SNP genotype and various clinico-pathological variables through case-only logistic regression analyses. The clinico-pathological variables evaluated were sex, age at CRC diagnosis (above or below the median age), tumour site (colon or rectum), stage at diagnosis (stage I/II or III/IV; presence or absence of distant metastasis) and family history of CRC (Table 4). No association was found between genotype and age at cancer diagnosis, site of cancer or a family history of CRC. The high-risk alleles of rs10795668 and rs4779584 were found to be significantly associated with male gender (*P*=0.03 and 0.01, respectively). Stratified analysis under additive model provided evidence that the association with CRC risk was limited to men for rs10795668, rs1295371 and rs4779584 (*P*=0.002, 0.002 and 0.002, respectively), whereas the association was limited to females for rs7014346 (*P*=0.011). A test for interaction of rs4779584 (using the additive model) with sex was statistically significant (*P*=0.038). There was no significant interaction of the other three SNPs with sex. Both rs12953717 and rs7014346 were associated with tumour stage. The high-risk allele of rs12953717 was significantly associated with stage IV at presentation (*P*=0.04), whereas the high-risk allele of rs7014346 was significantly associated with stage III or IV disease (*P*=0.01).

Of the four independent risk variants examined, none demonstrated statistically significant difference in effect size between the Hong Kong Han Chinese and the Caucasian European populations (Table 5). For rs7014346, there was also no difference in effect sizes among four populations (i.e. Hong Kong Han Chinese, Japanese, English and Scottish).

## Discussion

Although a number of CRC risk variants have now been identified, almost all have been through analyses based on European Caucasian populations. As the incidence of CRC and the allele frequencies of SNPs differ across populations, it is important to understand the effects of these markers in other populations. We, therefore, comprehensively examined the association between 32 SNPs and CRC risk and clinico-pathological variables in Chinese CRC patients recruited from hospitals across Hong Kong. Twelve SNPs from four independent susceptibility loci (at 8q24.21, 10p14, 15q14 and 18q21.2) were found to be significantly associated with CRC in the Han Chinese population in Hong Kong. A composite index of nine independent SNPs was significantly associated with CRC risk, which provides support for the CRC association findings in European populations. Although we recommend caution in implementing genetic models for predicting individual risk, approaches incorporating multilocus genotypes could help identify high-risk subgroups within a population. This underscores the potential for future risk profiling, even without identification of the causative variant (Wray *et al*, 2007). However, large multinational cohort studies will be needed to validate such genetic risk predictive models.

The rs10795668 provided the strongest evidence for an association in the Han Chinese population. This SNP maps to an 82-kb block of LD (8.73–8.81 Mb) within 10p14. All five additional SNPs, rs706771, rs827401, rs7894531, rs7898455 and rs4474353, mapping to this LD block showed evidence of association with CRC risk. The inclusion of each of these additional SNPs did not significantly improve the fit of the model compared with rs10795668 alone, providing no evidence for more than one disease locus at 10p14.

There are no proven protein-coding transcripts in the vicinity of the marker SNPs that we tested, and there is no predicted gene within 0.4 Mb of rs10795668. The nearest predicted genes are BC031880, located 0.4 Mb proximal to rs10795668, and LOC389935, located 0.7 Mb distally. Although loss of heterozygosity involving Chromosome 10p14 is seen in CRC (Shima *et al*, 2005), the underlying basis of the association identified at rs10795668 is presently unclear, but there is no evidence to implicate the predicted gene FLJ3802842 (Tomlinson *et al*, 2008). In the CEU population, there was some evidence that the effect of rs10795668 on CRC risk varied by the site of the tumour, with the susceptibility allele more common in rectal cancers (Tomlinson *et al*, 2008). This was not seen in the Han Chinese population we studied.

The SNP rs4779584 maps to Chr15:30 782 048, that is the CRAC (HMPS) locus. Although the risk allele in our population is the same as the European population, T is a major allele (0.83) in our population, whereas it is a minor allele in the CEU population (0.19). A previous meta-analysis by Jaeger *et al* (2008) showed a very strong association of rs4779584 with CRC risk. Two out of nine additional SNPs tested in this region were also statistically significant (rs16970016 and rs1554865) in our population; rs10318, which maps 31 kb distal to rs4779584, was one of the two most strongly associated SNPs in the CEU population; yet, such finding could not be replicated in our study. One of the possible reasons for this disparity is that there are differences at this locus between the CEU and CHB population in terms of LD structure (Supplementary Table 3a and b). For example, there are vast differences in the MAFs for rs10318 (CEU 0.18, CHB 0.49 and HK control 0.46). Differences in MAFs between the two populations and the nature of the minor alleles were also found for other SNPs tested in this study (MAF: rs6494587, rs1696968, rs11632715 and rs1406387; nature of minor alleles: rs1554865, rs16970016, rs1406389, rs1919360 and rs7165427).

In the European studies, no association was found between the genotypes of rs4779584 and any of the clinico-pathological variables tested. In the Han Chinese population, the risk allele of rs4779584 was significantly associated with the male gender. Moreover, there was significant interaction between this SNP and the gender. This SNP rs4779584 lies between GREM1 and SCG5. Jaeger *et al* (2008) have previously reported no association between SCG5 or GREM1 expression and the genotype of rs47795684. The GREM1 encodes a secreted bone morphogenetic protein (BMP) antagonist. The TGF-*β*/BMP pathway is known to have an important role in colorectal tumourigenesis. It is, therefore, plausible that GREM1 may increase tumour proliferation, for example, through its expression in the stroma (Sneddon *et al*, 2006). Although SCG5 is genetically and functionally slightly worse candidate than GREM1, neuroendocrine signalling involving SCG5 (Seidah and Chretien, 1999) could influence cellular proliferation in the large bowel through, for example, signalling of nutrient availability or systemic hormonal effect.

The SNP rs12953717 is located at intron 3 of the SMAD7 gene on 18q21. One of the other two SNPs (rs4939827) tested in this region was also statistically significant. Yet, the inclusion of rs4939827 did not improve the fit of the model compared with rs12953717 alone; such result was compatible with there being a single risk locus in the SMAD7 region. The risk allele, C, was a major allele in our study, whereas it was a minor allele in the CEU studies. Although 18q21.1 contains another protein-coding gene (CR621005) and a predicted gene of unknown function (KIA0427), the decay in LD away from SMAD7 intron 3 incorporating all three SNPs as shown by Broderick *et al* (2007) did not support these genes as the location of a causative variant.

Loss of chromosome 18q is very common in individuals with CRC. Broderick *et al* (2007) observed that lower median SMAD7 mRNA expression was associated with CRC risk allele at rs12953717. The SMAD7 acts as an intracellular antagonist of TGF-*β* signalling by binding stably to the receptor complex and blocking activation of downstream signalling events. Pertubation of SMAD7 expression has been documented to influence CRC progression (Levy and Hill, 2006) and SMAD7 has also been shown to induce hepatic metastasis in CRC (Halder *et al*, 2008). Our finding of significant association of rs129753717 with metastatic disease supports the observations that SMAD7 influences CRC progression and induces distant metastasis. In a recent study, Thompson *et al* (2009) had shown gender-specific association of SMAD7 with colon cancer risk (i.e. risk association in women only). However, in our study, stratified analysis revealed significant association of rs12975717 with CRC risk in man only, while the interaction between rs12975717 and gender was not significant. There is no obvious explanation for the disparity in these study findings.

Located on 8q24, rs7014346 is in strong LD with rs6983267; rs7014346 is 3 kb upstream of POU5F1P1 and maps within intron 6 of the gene DQ515897. Although this is close to genes encoding POU transcription factors, recent data suggest that the causal basis of the 8q24 association is rs6983267, which impacts on the differential expression of c.MYC through a long range *cis*-effect (Pomerantz *et al*, 2009; Tuupanen *et al*, 2009). Previous European studies did not find any interaction between various clinical variables with rs7014346 (Tenesa *et al*, 2008). The locus at 8q24.21 has been previously reported to influence the risk of adenomas as well as CRC (Tomlinson *et al*, 2007), suggesting that the 8q24.21 locus was involved in tumour initiation rather than progression. In our study, we found an association of rs7014346 with aggressive-advanced cancer raising the possibility that the 8q24.21 locus is also involved in tumour progression.

Twenty previously identified risk SNPs were not associated with CRC risk in the Chinese population. Although rs3802842 was significantly associated with CRC risk in various Caucasian populations, this association has not been replicated in the Japanese and Hong Kong Han Chinese populations. Several reasons exist for a failure to replicate findings. First, it could be that this study had insufficient power to detect the modest effect sizes of these SNPs. Second, for some non-replicated SNPs, there are differences in terms of the allele frequencies and LD patterns between the CEU and HCB/HK data. Third, the magnitude of the effect of a risk allele may differ between populations because of gene–gene or gene–environment interactions.

The study provides replication of four independent SNPs and suggests that there is a great deal of commonality in the aetiology of CRC across populations. This may not be entirely surprising for such high-frequency variants as these are likely to have quite ancient origins before ethnic diversification.

## Figures and Tables

**Table 1:** Characteristics of the colorectal cancer cases studied

**Case subject characteristics**	**Phase I**	**Phase 2**
Number	716	892
		
*Age at diagnosis (year)*		
Median (range, interquartile range)	68 (58–76, 18)	68 (58–76, 18)
Mean (range, s.d.)	66.75 (31–96, 12.25)	66.43 (31–96, 12.21)
		
*Sex* (%)		
Male	445 (62.2)	519 (58.2)
Female	271 (37.8)	373 (41.8)
		
*Tumour site* (%)		
Colon	444 (62.0)	549 (61.5)
Rectum	265 (37.0)	338 (37.9)
Both sites (synchronous)	7 (1.0)	5 (0.6)
		
*AJCC cancer stage* (%)		
Stage I	90 (12.6)	112 (12.6)
Stage II	220 (30.7)	276 (30.9)
Stage III	227 (31.7)	287 (32.2)
Stage IV	171 (23.8)	207 (23.2)
Not defined	8 (1.1)	10 (1.1)

Abbreviation: AJCC=American Joint Committee on Cancer.

**Table 2:** Allele frequency in CRC cases and controls and OR for SNPs studied

**SNP**	**Chromosome region^a^**	**Position (bp)^b^**	**Alleles^c^**	**High-risk allele in previous studies**	**MAF cases**	**MAF controls**	**OR heterozygous (95% CI)**	**OR homozygous (95% CI)**	*P-*value*
*(A) SNPs analysed in Phase 1*									
rs6983267	8q24.21	128 482 487	G, T	G	0.46	0.43	0.98 (0.77–1.24)	1.31 (0.97–1.77)	0.127
rs7014346	8q24.21	128 493 974	A, G	A	0.34	0.3	1.15 (0.92–1.43)	1.7 (1.17–2.49)	0.0091
rs10795668	10p14	8 741 225	A,G	G	0.32	0.38	0.74 (0.59–0.92)	0.65 (0.47–0.90)	0.0018
rs3802842	11q23.1	110 676 919	C, A	C	0.46	0.44	1.29 (1.02–1.64)	1.16 (0.86–1.56)	0.225
rs4444235	14q22.3	53 480 669	T, C	C	0.48	0.48	1.03 (0.81–1.32)	1.03 (0.77–1.38)	0.822
rs4779584	15q14	30 782 048	C, T	T	0.17	0.21	0.76 (0.60–0.95)	0.74 (0.43–1.26)	0.017
rs10318	15q14	30 813 271	C, T	T	0.46	0.44	1.11 (0.88–1.42)	1.21 (0.89–1.64)	0.213
rs9929218	16q22.1	67 378 447	A, G	G	0.24	0.24	0.97 (0.78–1.21)	1.05 (0.65–1.67)	0.965
rs4939827	18q21.2	44 707 461	T, C	T	0.38	0.35	1.12 (0.90–1.40)	1.45 (1.03–2.04)	0.037
rs12953717	18q21.2	44 707 929	T, C	T	0.38	0.33	1.1 (0.88–1.37)	1.64 (1.15–2.34)	0.015
rs4464148	18q21.2	44 713 030	C, T	C	0.09	0.07	1.21 (0.90–1.62)	4.11 (0.46–36.19)	0.108
rs10411210	19q12	38 224 140	T, C	C	0.18	0.18	0.99 (0.78–1.25)	0.92 (0.52–1.62)	0.809
rs355527	20p12.3	6 336 068	A, G	A	0.08	0.07	1.25 (0.93–1.69)	0.38 (0.10–1.46)	0.49
									
*(B) SNPs analysed in Phase 2*									
rs706771	10p14	8 736 452	A, G	—	0.45	0.49	0.89 (0.72–1.11)	0.73 (0.56–0.95)	0.022
rs827401	10p14	8 738 836	A, G	—	0.45	0.49	0.89 (0.72–1.11)	0.74 (0.57–0.96)	0.028
rs7894531	10p14	8 774 767	T, G	—	0.34	0.38	0.76 (0.62–0.93)	0.72 (0.54–0.96)	0.006
rs7898455	10p14	8 778 914	T, G	—	0.34	0.38	0.77 (0.63–0.94)	0.71 (0.53–0.95)	0.005
rs4474353	10p14	8 783 319	A, G	—	0.33	0.38	0.76 (0.62–0.94)	0.71 (0.54–0.95)	0.005
rs11623717	14q22.3	53 483 882	G, A	—	0.43	0.44	1.02 (0.83–1.26)	0.93 (0.71–1.21)	0.679
rs17563	14q22.3	53 487 272	C, T	—	0.29	0.27	1.08 (0.89–1.31)	1.32 (0.92–1.89)	0.142
rs2071047	14q22.3	53 488 161	T, C	—	0.41	0.41	1.03 (0.84–1.27)	0.97 (0.74–1.27)	0.9
rs2761887	14q22.3	53 494 802	C, A	—	0.44	0.44	1.0 (0.81–1.24)	0.99 (0.76–1.29)	0.961
rs8014363	14q22.3	53 501 325	C, T	—	0.1	0.11	0.99 (0.78–1.26)	0.61 (0.25–1.48)	0.584
rs6494587	15q14	30 768 935	A, G	—	0.42	0.4	1.17 (0.95–1.43)	1.16 (0.88–1.52)	0.194
rs16969681	15q14	30 780 403	T, C	—	0.36	0.34	1.15 (0.94–1.40)	1.16 (0.85–1.59)	0.185
rs16970016	15q14	30 782 590	C, A	—	0.22	0.25	0.81 (0.67–0.99)	0.72 (0.48–1.07)	0.017
rs1554865	15q14	30 787 098	A, G	—	0.21	0.25	0.83 (0.68–1.01)	0.68 (0.45–1.02)	0.015
rs11632717	15q14	30 791 539	G, A	—	0.23	0.23	1.0 (0.82–1.21)	1.22 (0.76–1.94)	0.644
rs1406389	15q14	30 796 770	T, A	—	0.28	0.3	0.94 (0.77–1.15)	0.78 (0.56–1.09)	0.173
rs1919360	15q14	30 830 747	C, T	—	0.38	0.37	0.96 (0.79–1.18	1.05 (0.78–1.41)	0.91
rs7165427	15q14	30 947 552	T, C	—	0.5	0.5	0.89 (0.71–1.12)	0.99 (0.76–1.29)	0.946
rs961253	20p12.3	6 352 281	A, C	—	0.08	0.074	1.28 (0.98–1.67)	0.34 (0.09–1.26)	0.337

Abbreviations: CI=confidence intervals; CRC=colorectal cancer; MAF=minor allele frequency; OR=odds ratio; SNP=single-nucleotide polymorphism.

a Chromosomal regions were obtained from NCBI map viewer, Ideogram section.

b All base pairs were according to the NCBI build 36.3 data.

c Order of allele: minor allele, major allele; the allele underlined denoted high-risk allele in our study.

OR heterozygous – OR in heterozygotes, relative to common homozygotes.

OR homozygous – OR in rare homozygotes, relative to common homozygotes.

* *P-*values were obtained by Cochrane–Armitage trend test with one degree of freedom; *P-*values obtained from logistic regression models were similar (data not shown). In Phase 1, if an OR had the same direction as previously reported, one-tailed *P-*values were calculated, i.e. *P*/2, conversely for ORs in the opposite direction *P*=1−*P*/2.

**Table 3:** Association between total number of risk alleles and colorectal cancer risk

**Total no. of risk alleles**	**Cases No. (%)**	**Controls No. (%)**	**OR (95% CI)**	
4–6	25 (3.6)	42 (5.9)	0.54 (0.32–0.94)	
7	39 (5.6)	76 (10.7)	0.47 (0.30–0.74)	
8	101 (14.4)	131 (18.5)	0.70 (0.50–1.00)	
9	160 (22.8)	128 (18.1)	1.14 (0.82–1.59)	
10	151 (21.4)	138 (19.5)	1.0 (reference)	
11	110 (15.7)	104 (14.7)	0.97 (0.68–1.38)	
12	77 (11.0)	61 (8.5)	1.15 (0.77–1.73)	
13–15	39 (5.5)	29 (4.1)	1.23 (0.72–2.09)	
				*P*_trend_=2.29 × 10^−5^

Abbreviations: CI=confidence intervals; OR=odds ratio; SNP=single-nucleotide polymorphism.

SNPs used to calculate number of risk alleles: rs7014346, rs10795668, rs3802842, rs4444235, rs4779584, rs9929218, rs12953717, rs10411210 and rs355527.

High-risk allele: more frequent allele observed in cases from reference study.

*P* – obtained from *χ*^2^ test.

*P*_trend_ – obtained from Cochrane–Armitage trend test.

**Table 4:** Association of independent SNPs with various clinico-pathological variables

		**Sex: male (M), female (F)**			**Age: <median, ⩾median**			**Cancer stage: stage I/II, stage III/IV**			**Metastatic disease: not stage IV, stage IVs**			**Family history of CRC**			**Site of cancer colon (C) rectum (R)**		
**SNP**	**Genotype**	**M**	**F**	***P***	**<68**	**⩾68**	***P***	**I and II**	**III and IV**	***P***	**Not IV**	**IV**	***P***	**Yes**	**No**	***P***	**C**	**R**	***P***
rs10795668	AA	54	55		49	60		42	64		84	22		3	106		73	33	
Risk allele: G	AG	209	161	0.03	192	178	0.57	157	210	0.17	278	89	0.99	36	334	0.21	222	145	0.50
	GG	244	156		185	215		184	213		308	89		36	364		247	151	
rs4779584	CC	14	12		9	17		15	11		22	4		4	22		15	11	
Risk allele: T	CT	107	87	0.01	87	107	0.12	80	111	0.64	142	49	0.88	16	178	0.39	126	65	0.71
	TT	324	172		248	248		215	276		373	118		42	454		303	189	
rs12953717	TT	60	37		51	46		45	51		66	30		5	92		65	32	
Risk allele: T	TC	220	123	0.48	168	175	0.21	145	194	0.77	255	84	0.04	43	300	0.22	231	125	0.24
	CC	165	111		125	151		120	153		216	57		14	262		166	108	
rs7014346	AA	50	31		39	42		32	49		60	21		7	74		56	25	
Risk allele: A	AG	199	132	0.39	162	169	0.65	128	199	0.01	237	90	0.06	33	298	0.35	205	123	0.26
	GG	196	108		143	161		150	150		240	60		22	282		183	117	

Abbreviations: CRC=colorectal cancer; SNP=single-nucleotide polymorphism.

*P* – *P-*values obtained from logistic regression model.

**Table 5:** Heterogeneity of associations between Hong Kong Han Chinese population and other populations

	**OR (95% CI), high-risk allele frequency in control subjects; ref allele: low-risk allele in this study**				**Breslow–Day's test *P*_heterogeneity_ with this study**			
**SNP, chromosome**	**HK**	**England/CEU**	**Japan**	**Scotland**	**England/CEU**	**Japan**	**Scotland**	**Reference**
rs7014346, 8q24.21	1.23 (1.05–1.44), 30%	1.29 (1.18–1.40), 36%	0.85 (0.79–0.92), 77%^a^	1.23 (1.15–1.33), 37%	0.60	3.67 × 10^–5^	0.95	Tenesa *et al* (2008)
rs10795668, 10p14	1.28 (1.1–1.5), 62%	1.12 (1.00–1.25), 67%	—		0.17			Tomlinson *et al* (2008)
rs4779584, 15q14	1.26 (1.04–1.52), 79%	1.21 (1.12–1.31), 19%^a^	—		0.72			Jaeger *et al* (2008)
rs12953717, 18q21.2	1.20 (1.03–1.40), 33%	1.38 (1.21–1.56), 42%	—		0.19			Broderick *et al* (2007)
rs4939827, 18q21.2	1.17 (1.01–1.37), 35%	1.40 (1.23–1.59), 51%^a^	—		0.08			Broderick *et al* (2007)
rs10318, 15q14	1.10 (0.95–1.27), 44%	0.79 (0.67–0.94), 82%^a^	—		0.005			Jaeger *et al* (2008)
rs4464148, 18q21.2	1.20 (1.03–1.4), 33%	1.35 (1.18–1.55), 29%	—		0.29			Broderick *et al* (2007)
rs12953717, 15q14	1.2 (1.03–1.4), 33%	1.38 (1.21–1.56), 42%	—		0.19			Broderick *et al* (2007)
rs6983267, 8q24.21	1.12 (0.97–1.3), 43%	1.41 (1.24–1.6), 49%	—		0.02			Tomlinson *et al* (2007)
rs4444235, 14q22.3	1.02 (0.88–1.18), 48%	0.89 (0.85–0.93), 54%^a^	—		0.21			Houlston *et al* (2008)
rs9929218, 16q22.1	1.00 (0.84–1.18), 76%	1.14 (1.08–1.21), 70%	—		0.15			Houlston *et al* (2008)
rs355527, 20p12.3	1.10 (0.84–1.45), 7%	1.13 (1.08–1.19), 33%	—		0.85			Houlston *et al* (2008)
rs10411210, 19q12	1.02 (0.85–1.24), 82%	1.27 (1.16–1.38), 90%	—		0.045			Houlston *et al* (2008)

Abbreviations: CEU=Caucasian European; CI=confidence interval; CRC=colorectal cancer; HK=Hong Kong; OR=odds ratio; SNP=single-nucleotide polymorphism.

a High-risk allele different in this and reference study.
