# Supplementary material for: Replication study of SNP associations for colorectal cancer in Hong Kong Chinese
Source: Br J Cancer. 2010 Dec 21;104(2):369–75. doi: 10.1038/sj.bjc.6605977 (PMC3031883; doi:10.1038/sj.bjc.6605977)
Supplement: Supplementary Tables 1–3 [file 6605977x1.doc]

Supplementary table 1a: Genotype counts and minor allele in phase one

| Chr | SNP | Allele1 (A1) | Allele2 (A2) | A1A1/A1A2/A2A2 Case | A1A1/A1A2/A2A2 Control |
| --- | --- | --- | --- | --- | --- |
| 8 | rs6983267 | G | T | 155/346/215 | 123/367/223 |
| 8 | rs7014346 | A | G | 81/331/304 | 53/322/339 |
| 10 | rs10795668 | A | G | 84/294/333 | 107/329/275 |
| 11 | rs3802842 | C | A | 145/374/197 | 146/338/230 |
| 14 | rs4444235 | T | C | 170/350/195 | 168/346/199 |
| 15 | rs4779584 | C | T | 26/194/496 | 32/232/450 |
| 15 | rs10318 | C | T | 143/371/202 | 129/363/220 |
| 16 | rs9929218 | A | G | 39/265/410 | 37/270/407 |
| 18 | rs4939827 | T | C | 100/348/268 | 76/342/296 |
| 18 | rs12953717 | T | C | 97/343/276 | 65/345/304 |
| 18 | rs4464148 | C | T | 4/114/598 | 1/97/615 |
| 19 | rs10411210 | T | C | 24/204/485 | 26/205/482 |
| 20 | rs355527 | A | G | 3/110/601 | 8/90/616 |
|  |  |  |  |  |  |
| Supplementary table 1b: Genotype counts and minor allele in phase two | | | | | |
| Chr | SNP | Allele1 (A1) | Allele2 (A2) | A1A1/A1A2/A2A2 Case | A1A1/A1A2/A2A2 Control |
| 10 | rs706771 | A | G | 178/444/266 | 216/441/236 |
| 10 | rs827401 | A | G | 177/443/268 | 212/442/238 |
| 10 | rs10795668 | A | G | 109/370/400 | 134/411/341 |
| 10 | rs7894531 | A | G | 113/373/401 | 133/415/341 |
| 10 | rs7898455 | T | G | 113/374/402 | 135/414/343 |
| 10 | rs4474353 | A | G | 111/371/405 | 133/414/344 |
| 14 | rs11623717 | G | A | 167/430/291 | 179/421/291 |
| 14 | rs17563 | C | T | 76/361/450 | 61/354/476 |
| 14 | rs2071047 | T | C | 151/427/311 | 158/420/315 |
| 14 | rs2761887 | C | A | 179/429/281 | 181/430/282 |
| 14 | rs8014363 | C | T | 8/163/720 | 13/163/715 |
| 15 | rs6494587 | A | G | 157/431/299 | 150/409/331 |
| 15 | rs16969681 | T | C | 100/432/357 | 94/410/389 |
| 15 | rs16970016 | C | A | 47/288/554 | 60/325/508 |
| 15 | rs1554865 | A | G | 45/284/558 | 61/317/515 |
| 15 | rs11632715 | G | A | 42/328/518 | 35/333/525 |
| 15 | rs1406389 | T | A | 74/340/474 | 91/346/455 |
| 15 | rs1919360 | C | T | 124/418/347 | 117/431/344 |
| 15 | rs7165427 | T | C | 221/442/225 | 211/468/213 |
| 20 | rs961253 | A | C | 3/141/744 | 9/114/768 |

Supplementary table 2a: Logistic regression analyses of independent SNPs within each LD region in phase one.

| CHR | Other SNP | BP | Most Significant SNP | *P*-value# |
| --- | --- | --- | --- | --- |
| 8 | rs6983267 | 128482487 | rs7014346 | 0.43 |
| 15 | rs10318 | 30813271 | rs4779584 | 0.06 |
| 18 | rs4939827 | 44707461 | rs12953717 | 0.20 |
| 18 | rs4464148 | 44713030 | rs12953717 | 0.37 |

Supplementary table 2b: Logistic regression analyses of independent SNPs within each LD region in phase two.

| CHR | Other SNP | BP | Most Significant SNP | *P*-value# |
| --- | --- | --- | --- | --- |
| 10 | rs706771 | 8736452 | rs7898455 | 0.89 |
| 10 | rs827401 | 8738836 | rs7898455 | 0.97 |
| 10 | rs7894531 | 8774767 | rs7898455 | NA |
| 10 | rs4474353 | 8783319 | rs7898455 | NA |
| 10 | rs706771 | 8736452 | rs4474353 | 0.94 |
| 10 | rs827401 | 8738836 | rs4474353 | 0.93 |
| 10 | rs7894531 | 8774767 | rs4474353 | 0.68 |
| 10 | rs7898455 | 8778914 | rs4474353 | NA |
| 15 | rs6494587 | 30768935 | rs1554865 | 0.47 |
| 15 | rs16969681 | 30780403 | rs1554865 | 0.59 |
| 15 | rs16970016 | 30782590 | rs1554865 | 0.73 |
| 15 | rs11632715 | 30791539 | rs1554865 | 0.22 |
| 15 | rs1406389 | 30796770 | rs1554865 | 0.67 |
| 15 | rs1919360 | 30830747 | rs1554865 | 0.54 |
| 15 | rs7165427 | 30947552 | rs1554865 | 0.96 |

# *P*-value: significant *p*-value suggests that the other SNP have independent effect.

Supplementary table 2c: Pairwise LD measures for multiple SNPs pairs (Phase 2, Chromosome 10)

| SNP1 | SNP2 | D' | r^2^ |
| --- | --- | --- | --- |
| rs706771 | rs827401 | 1 | 1 |
| rs706771 | rs10795668 | 1 | 0.76 |
| rs706771 | rs7894531 | 0.97 | 0.65 |
| rs706771 | rs7898455 | 0.97 | 0.63 |
| rs706771 | rs4474353 | 0.97 | 0.63 |
| rs827401 | rs10795668 | 1 | 0.76 |
| rs827401 | rs7894531 | 0.97 | 0.65 |
| rs827401 | rs7898455 | 0.97 | 0.63 |
| rs827401 | rs4474353 | 0.97 | 0.63 |
| rs10795668 | rs7894531 | 0.98 | 0.86 |
| rs10795668 | rs7898455 | 0.97 | 0.84 |
| rs10795668 | rs4474353 | 0.97 | 0.84 |
| rs7894531 | rs7898455 | 1 | 0.98 |
| rs7894531 | rs4474353 | 1 | 0.98 |
| rs7898455 | rs4474353 | 1 | 1 |

Supplementary table 3a: Minor allele and minor allele frequencies (MAF) in different population in phase one

| SNP | chromosomal regions ♦ | Position + | Allele1 | Allele2 | Minor allele from Hapmap | | MAF | |
| --- | --- | --- | --- | --- | --- | --- | --- | --- |
|  |  |  |  |  | CEU | HCB | CEU | HCB |
| rs10795668 | 10p14 | 8741225 | A | G | A | A | 0.36 | 0.35 |
| rs3802842 | 11q23.1 | 110676919 | C | A | C | C | 0.24 | 0.38 |
| rs4444235 | 14q22.3 | 53480669 | T | C | C | T | 0.43 | 0.48 |
| rs4779584 | 15q14 | 30782048 | C | T | T | C | 0.16 | 0.20 |
| rs10318 | 15q14 | 30813271 | C | T | T | T | 0.18 | 0.49 |
| rs9929218 | 16q22.1 | 67378447 | A | G | A | A | 0.26 | 0.18 |
| rs4939827 | 18q21.2 | 44707461 | T | C | C | T | 0.49 | 0.26 |
| rs12953717 | 18q21.2 | 44707927 | T | C | C | T | 0.24 | 0.18 |
| rs4464148 | 18q21.2 | 44713030 | C | T | C | C | 0.30 | 0.03 |
| rs10411210 | 19q12 | 38224140 | T | C | T | T | 0.11 | 0.19 |
| rs355527 | 20p12.3 | 6336068 | A | G | A | A | 0.34 | 0.06 |
| rs6983267 | 8q24.21 | 128482487 | G | T | G | G | 0.46 | 0.39 |
| rs7014346 | 8q24.21 | 128493974 | A | G | A | A | 0.27 | 0.36 |

Supplementary table 3b: Minor allele and minor allele frequencies (MAF) in different population in phase two

| SNP | chromosomal regions ♦ | Position + | Allele1 | Allele2 | Minor allele from Hapmap | | MAF | |
| --- | --- | --- | --- | --- | --- | --- | --- | --- |
|  |  |  |  |  | CEU | HCB | CEU | HCB |
| rs706771 | 10p14 | 8736452 | A | G | A | A | 0.37 | 0.41 |
| rs827401 | 10p14 | 8738836 | A | G | A | A | 0.34 | 0.41 |
| rs10795668 | 10p14 | 8741225 | A | G | A | A | 0.36 | 0.35 |
| rs7894531 | 10p14 | 8774767 | A | G | A | A | 0.36 | 0.36 |
| rs7898455 | 10p14 | 8778914 | T | G | T | T | 0.38 | 0.36 |
| rs4474353 | 10p14 | 8783319 | A | G | A | A | 0.36 | 0.36 |
| rs11623717 | 14q22.3 | 53483882 | G | A | G | G | 0.40 | 0.41 |
| rs17563 | 14q22.3 | 53487272 | C | T | T | C | 0.39 | 0.32 |
| rs2071047 | 14q22.3 | 53488161 | T | C | T | T | 0.38 | 0.42 |
| rs2761887 | 14q22.3 | 53494802 | C | A | C | C | 0.45 | 0.41 |
| rs8014363 | 14q22.3 | 53501325 | C | T | C | C | 0.45 | 0.11 |
| rs6494587 | 15q14 | 30768935 | A | G | A | A | 0.07 | 0.41 |
| rs16969681 | 15q14 | 30780403 | T | C | T | T | 0.03 | 0.20 |
| rs16970016 | 15q14 | 30782590 | C | A | A | C | 0.13 | 0.22 |
| rs1554865 | 15q14 | 30787098 | A | G | G | A | 0.15 | 0.24 |
| rs11632715 | 15q14 | 30791539 | G | A | A | G | 0.45 | 0.24 |
| rs1406389 | 15q14 | 30796770 | T | A | A | T | 0.16 | 0.35 |
| rs1919360 | 15q14 | 30830747 | C | T | T | C | 0.15 | 0.44 |
| rs7165427 | 15q14 | 30947552 | T | C | C | C | 0.42 | 0.47 |
| rs961253 | 20p12.3 | 6352281 | A | C | A | A | 0.37 | 0.05 |

♦ - chromosomal regions were obtained from NCBI map viewer, Ideogram section

+ - all bp were according to the NCBI build 36.3 data

MAF – minor allele frequency from Hapmap
